# The SmokefreeTXT (SFTXT) Study: Web and Mobile Data Collection to Evaluate Smoking Cessation for Young Adults

**DOI:** 10.2196/resprot.5653

**Published:** 2016-06-27

**Authors:** Linda Squiers, Derick Brown, Sarah Parvanta, Suzanne Dolina, Bridget Kelly, Jill Dever, Brian G Southwell, Amy Sanders, Erik Augustson

**Affiliations:** ^1^ RTI International Center for Communication Science Rockville, MD United States; ^2^ RTI International Research Triangle Park, NC United States; ^3^ RTI International Washington, DC United States; ^4^ ICF International Rockville, MD United States; ^5^ National Cancer Institute Division of Cancer Control and Population Sciences Rockville, MD United States

**Keywords:** tobacco use cessation, text messaging, clinical trial, survey methods, cell phones, telemedicine

## Abstract

**Background:**

Text messaging (short message service, SMS) has been shown to be effective in delivering interventions for various diseases and health conditions, including smoking cessation. While there are many published studies regarding smoking cessation text messaging interventions, most do not provide details about the study’s operational methods. As a result, there is a gap in our understanding of how best to design studies of smoking cessation text messaging programs.

**Objective:**

The purpose of this paper is to detail the operational methods used to conduct a randomized trial comparing three different versions of the National Cancer Institute’s SmokefreeText (SFTXT) program, designed for smokers 18 to 29 years of age. We detail our methods for recruiting participants from the Internet, reducing fraud, conducting online data collection, and retaining panel study participants.

**Methods:**

Participants were recruited through website advertisements and market research online panels. Screening questions established eligibility for the study (eg, 18 to 29 years of age, current smoker). Antifraud measures screened out participants who could not meet the study requirements. After completing a baseline survey, participants were randomized to one of three study arms, which varied by type and timing of text message delivery. The study offered US $20 gift cards as incentives to complete each of four follow-up surveys. Automated email reminders were sent at designated intervals to increase response rates. Researchers also provided telephone reminders to those who had not completed the survey after multiple email reminders. We calculated participation rates across study arms and compared the final sample characteristics to the Current Population Survey to examine generalizability.

**Results:**

Recruitment methods drove 153,936 unique visitors to the SFTXT Study landing page and 27,360 began the screener. Based on the screening questions, 15,462 out of 27,360 responders (56.51%) were eligible to participate. Of the 15,462 who were eligible, 9486 passed the antifraud measures that were implemented; however, 3882 failed to verify their email addresses or cell phone numbers, leaving 5604 who were invited to complete the baseline survey. Of the 5604 who were invited, 4432 completed the baseline survey, but only 4027 were retained for analysis because 405 did not receive the intervention.

**Conclusions:**

Although antifraud measures helped to catch participants who failed study requirements and could have biased the data collected, it is possible that the email and cell phone verification check excluded some potentially eligible participants from the study. Future research should explore ways to implement verification methods without risking the loss of so many potential participants.

**ClinicalTrial:**

Clinical Trials.gov NCT01885052; https://clinicaltrials.gov/ct2/show/NCT01885052; (Archived by WebCite at http://www.webcitation.org/6iWzcmFdw)

## Introduction

Text messaging (short message service, SMS) has been shown to be effective in delivering interventions for various diseases and health conditions, including smoking cessation. There are many similarities in the design of existing text-based cessation interventions. Kong et al [1] conducted a narrative review of 15 text-based smoking cessation interventions and found that all based their motivational messages in cognitive behavioral and social cognitive theories focused on self-efficacy, with roughly half of the interventions tailoring the message content according to baseline questionnaire responses. All studies recruited participants who indicated that they were willing to quit smoking, and seven of the programs recruited young adults 18 to 29 years of age. The reviewed interventions varied in duration and format. All interventions included text messages to participants during the active quit phase, which ranged from 1 to 13 weeks; 10 interventions also included a preparation phase (ranging from 1 to 4 weeks) before the participant reached his or her quit date, and 8 included a maintenance phase during which the number of text messages received decreased over time. Similarly, the number of text messages varied by intervention, from nine messages per day to three per week, with increased frequency during the active quit period. Eleven of the interventions reviewed specifically offered or informed participants about other cessation treatments, including support through email, websites, self-help booklets, and medication, to supplement the text messaging intervention.

When developing smoking cessation studies, researchers also look to previous studies to answer questions pertaining to how study participants should be recruited, what type of data collection strategy would work best for a smoking cessation text messaging program, how frequently the data should be collected, what kind of incentive (if any) should be used, what type of antifraud methods should be used, how long recruitment should last, what response and retention rates can be anticipated, and what level of follow-up is required to retain participants through all data collection activities.

While the review by Kong et al provided details about the key elements of text messaging programs, it did not provide clear answers to these specific issues; however, it did synthesize the general methods used in the 15 studies that were reviewed. This review indicated that recruitment strategies for text-based cessation interventions most frequently used online venues (eg, Google, Facebook, Craigslist; [2-4]), although Free et al [5] reported use of a wide variety of recruitment methods, including radio, billboards, newspapers, and cessation service providers. Kong et al found that data collection tended to be conducted online [3-5] with secondary options including phone [5] and text message [4]. Very few studies reported offering a financial incentive for data collection efforts. Ybarra et al [4] offered US $10 to $20 for completion of post-quit follow-up surveys, but they did not discuss fraud that may have occurred as a result of this financial incentive. Reviews and individual studies often do not provide the details about the operational methods used, and do not highlight the lessons learned from using these methods, resulting in a gap in our understanding of how best to design studies of smoking cessation text messaging programs.

### Study Objective

While some studies provide a general description of study methods, most do not provide the details about operational methods used to conduct a study. The purpose of this paper is to detail the operational methods we used to conduct a randomized trial comparing three different versions of National Cancer Institute’s (NCI) SmokefreeText (SFTXT) program (Clinical Trials.gov NCT01885052). In particular, we detail our methods for recruiting participants from the Internet, reducing fraud, conducting online data collection, and maintaining the panel of over 4000 study participants.

### Rationale for the Study

This study was developed to compare three different versions of NCI’s SFTXT program, which is designed for smokers 18 to 29 years of age. The current SFTXT program (available at NCI’s smokefree.gov website [6]) is an 8-week text messaging program that includes 2 weeks of preparatory messages before a participant’s quit date and 6 weeks of motivational support messages after a participant’s quit date. For this study, we assigned a quit date 2 weeks post-baseline, as opposed to the real-life application of SFTXT that allows users to choose their own quit date. Program data indicated that 34.1% of participants dropped out of the program within one week after their chosen quit date. Given these data, NCI was interested in determining whether a modified program focusing only on preparatory messages and quit-day support messages would be as effective as the full program. NCI also wanted to determine how these high-intensity motivational messaging programs compared with a low-intensity program that provided only quit date reminders and smoking status check-in messages. Three different versions of the SFTXT program were tested using a randomized three-arm longitudinal study design. The number of texts received varied by study arm, with Arm 1 participants receiving 11, Arm 2 participants receiving 40, and those in Arm 3 receiving a total of 127 text messages. Participants received a variety of types of texts depending on their group assignment. Examples of the types of texts included quit date reminders, tips on staying smoke-free, motivational messages, facts about smoking cessation, mood assessments, and others. The authors can be contacted for a full description of the study, including details about the intervention components of all three SFTXT programs tested.

## Methods

### Recruitment Goal and Power

To determine the sample size needed to conduct this study, we performed a power analysis [7] assuming a 32% predicted smoking abstinence rate and a 60% wave-to-wave attrition rate. The analytic goal was to detect a 5% difference between any two arms within the experimental design with 80% power and a two-sided test. This power analysis yielded a conservative baseline recruitment size of 4248 (1416 per arm) and a minimum sample size of 435 participants per arm to complete all five surveys.

### Recruitment Methods

#### Advertisements

SFTXT Study participants were recruited from June 26, 2013, through January 8, 2014, using a series of online strategies. Advertisements were posted on Facebook, Craigslist, and Pandora, along with search ads on Google, Yahoo!, and Bing. Additionally, market research firms emailed announcements about the study to their online panel members who smoked, and were within the eligible age group. Individuals who clicked on an SFTXT Study advertisement were directed to a 10-question screener to determine study eligibility.

#### Eligibility Criteria

To be eligible to participate in the study, individuals had to (1) be aged 18 to 29, (2) live in the United States, (3) have smoked on at least 5 of the past 30 days, (4) be at least moderately interested in stopping smoking within the next 30 days, and (5) not be seeking cessation services elsewhere. Given that all study communication was conducted via email or by text message, participants needed to have an active email address and agree to receiving up to 130 messages over 8 weeks on their mobile phones. Only one family member per household was eligible to participate in the study, as determined by a question on the screener questionnaire, and verified using an automated Internet Protocol (IP) address duplication check. Participants could not have a close friend who was already participating in the study. Finally, to be eligible, individuals had to be willing to share their contact information.

Eligible individuals completed an online consent form and were required to provide their email address (entered twice) and mobile phone number, and give permission for the study team to use the email and phone number to send surveys and leave reminder messages. Eligible individuals were also asked for an alternate phone number, but could participate in the study without providing one. Ineligible individuals were asked to complete a four-question exit survey that included demographic questions about sex, race/ethnicity, and education level.

#### Email and Text Verification

To confirm contact information, participants responded to an email verification request and a text message verification request before receiving the baseline survey. This procedure also verified that a participant could receive both emails and text messages for the study.

#### Incentives

To keep participants engaged in the study, we initially offered a choice of an Amazon or iTunes electronic gift card as an incentive for completing each of the five surveys. We conducted an initial pilot study for 2 weeks in July, 2012. Within the first few days of this pilot, we noticed that similar email addresses were being used to enroll participants. For example, an enrollee might have entered Jane.Doe@gmail.com, and shortly thereafter we would find a similar email address, such as JDoe@gmail.com. Upon investigation, we discovered that the system we created to check for duplicate emails or phone numbers was not working properly. To verify that the second case was associated with the first, we examined the IP addresses from the two cases to see if they were identical. We identified 105 duplicate or fraudulent enrollees, and these cases were notified by email that they were terminated from the study. We stopped the study for 16 days to fix the system for duplication checks, and added more antifraud measures, which are described below. We also decided to stop incentivizing the baseline survey. Instead, we only incentivized the four follow-up surveys at US $20 per survey.

#### Antifraud Measures

To ensure the integrity of the SFTXT Study sample, multiple antifraud measures were implemented within the screener instrument to prevent two types of behaviors: (1) eligible participants enrolling multiple times in the SFTXT Study (presumably) to obtain multiple incentives (US $20 for completion of each of the four follow-up surveys), and (2) previously ineligible individuals reenrolling and changing their responses to be within the eligibility criteria.

Our antifraud process included the following measures:

- *CAPTCHA*. CAPTCHA is a technique used to verify that a person, and not a computer, is accessing the website. A common type of CAPTCHA asks a user to type letters or numbers that appear in a distorted image on the screen. This task is very difficult for a computer to perform, making it an effective way to verify that a person is on the other end of the transaction.
- *Honesty pledge.* An honesty pledge can be implemented at the start of a survey to attempt to improve data quality. Users are asked to acknowledge that they intend to answer the survey truthfully. Results from a recent study presented at the American Association of Public Opinion Reporting (AAPOR) suggested that these types of pledges can help reduce the occurrence of straightlining, question skipping, and other types of fraud [8].
- *Hosting the survey on a secure site*. With a secure site, only those with a password can gain access. This mechanism reduces the likelihood that a participant could tell their friends to take the survey to collect incentives. To access each survey, participants needed to enter the username and password that was sent to them with the link to the follow-up surveys.
- *Conducting rigorous data cleaning*. The cleaning process looked for straightlining, anyone completing the survey in an extremely short amount of time, and other such indicators.
- *Duplication checks*. The antifraud process included automated duplication checks of phone numbers, email addresses, and IP addresses. If duplicates were detected, the individual was excluded from the study. To ensure that no lapses occurred in antifraud prevention, retroactive duplication checks were implemented by linking cases to one another through the contact information and IP addresses, which helped to ensure the sample’s validity.

### Data Collection

Once participants completed the verification process, they were invited to participate in the study. Study data were collected at five time points: baseline, 3 weeks post-baseline (7 days post-quit date), 8 weeks post-baseline (6 weeks post-quit date), 20 weeks post-baseline (18 weeks post-quit date), and 32 weeks post-baseline (30 weeks post-quit date). Additionally, upon completion of the baseline survey, participants were randomly assigned to one of the three study arms. A quit date was set at 2 weeks after completion of the baseline survey for all study participants.

Participants were invited to complete each survey via an email with a link to the study website. Participants could complete the survey at one time or could save their partial responses and complete the survey later. Participants were required to take the baseline and 3-week follow-up survey within 14 days of the initial email invitation and within 36 days for the 8-week, 20-week, and 32-week surveys, and were classified as survey non-respondents if they did not do so. These participants, however, remained in the study and were invited to complete the remaining questionnaires. Participants who had not completed a survey were emailed reminders on the third, fourth, fifth, sixth, and tenth day after the invitation was sent. If a participant still had not completed a survey, a staff member telephoned them as a final reminder before expiration of the 2-week window for taking the survey.

### Panel Support and Maintenance

To support the study, project team members answered participants’ questions about the study via email and telephone. The majority of participant questions focused on the verification process, missing login information, and requests to have incentive gift codes re-sent. This process enabled participants to update their contact information and receive answers to questions regarding existing survey invitations and incentives, which helped to ensure that the intervention was implemented as designed.

Participants were allowed to withdraw from all or parts of the SFTXT Study at any time, including the 2-week period before the assigned quit date. Participants who texted “STOP” in the 2 weeks before the assigned quit date were opted out of the study entirely and received no future text messages or follow-up survey invitations, as they would not have received the intervention. Participants who texted “STOP” after the assigned quit date no longer received the text messages but continued to receive follow-up survey invitations and were still considered study participants. These individuals could still opt out entirely if they contacted the study director to leave the study completely.

### Statistical Analysis

To be considered a study participant, an individual needed to meet all eligibility criteria, complete the verification process, and complete the baseline survey. Individuals were defined as *verified* if they provided informed consent, pledged to give honest responses, provided an email address and phone number, and verified their contact information. For the baseline survey to be considered complete, individuals needed to respond to essential questions (eg, smoking history, demographics). To assess differences between arms for response and retention rates, we conducted significance testing based on a chi-squared test for differences between Arms 1 and 2, Arms 1 and 3, and Arms 2 and 3. To calculate the response rate for the baseline survey, we used the AAPOR [9] RR6 formula with *noncontact* and *other* set to zero.

## Results

### Participant Recruitment

Participants were recruited over 196 days. Figure 1 presents the steps involved in recruiting and determining the eligibility of study participants, and the number retained and excluded at each step, beginning with initial visits to the SFTXT landing page where the study screener was located. There were 153,936 unique visitors to the landing page, or 785 visits per day on average.

Of those 153,936 unique visitors, 27,360 (17.77%) began the screener. Based on their responses to the screener, 15,462 of 27,360 visitors (56.51%) met the study’s eligibility requirements. Failure to provide informed consent excluded 3428 of the 15,462 eligible participants (22.17%). Antifraud measures excluded 2548 of 12,034 participants who provided informed consent (21.17%): 576 (4.79%) refused the honesty pledge, 1312 (10.90%) did not provide contact information, and 660 (5.48%) failed the duplication checks, leaving 9486 of the 15,462 eligible participants (61.35%) who had both consented and passed the antifraud measures. Another 3882 of 9486 (40.92%) eligible participants were sent a verification code; however, they failed to verify their email address and phone number and were excluded based on this requirement.

The remaining 5604 respondents were sent an invitation to complete the baseline survey; 1172 of 5604 respondents (20.91%) did not complete this survey and were excluded from the study. Participants (405 of 4432, 9.14%) were also excluded if they did not receive the intervention. For example, 233 of 4432 faced technical difficulties (eg, undelivered text messages). Participants who texted “STOP” at any point *before* their quit date (n=150) were excluded because they had not received an essential part of the intervention (ie, messages on their quit date) and, depending on the study arm, may have received less than half of the text messages. A small number of participants (n=22) opted out of the study entirely by notifying the project team, typically via email. Those who texted “STOP” after the quit date were retained in the analytic sample.

### Final Sample

A total of 4,027 participants were retained for analysis, including participants who texted STOP *after* their quit date (n=236), as they had received the essential quit date messages and most of the intervention at that point. The frequency of texting “STOP” messages sent after the quit date was significantly higher in Arm 3 (153/236) than in Arm 1 (54/236) or Arm 2 (29/236) and significantly higher in Arm 1 than in Arm 2. Of these participants, 41 responded to a survey question asking why they opted out of receiving further text messages, with the majority (51%, 21/41) reporting that there were too many texts or that the texts were bothersome. Others stated that the texts did not help them quit smoking (10/41), or that they had stopped trying to quit (6/41).

### Response and Retention Rates

Verified and consented individuals (5604 of 9486 eligible participants) were invited to take the SFTXT baseline survey, and 4027 participants subsequently completed it and were retained for analysis (see Table 1). This sample size, which excludes participants who completed the baseline survey but were ultimately dropped from the analytic sample, equates to a response rate of 71.86% (using the AAPOR RR5 formula [8]).

**Table 1:** Response rates by recruitment stage and study arm (n=153,936).

Response rate for recruitment stage	Overall		Arm 1		Arm 2		Arm 3	
	n	%	n	%	n	%	n	%
Unique visits to project websites	153,936		—		—		—	
Initiated screener/initiation rate	27,630	17.77	—		—		—	
Completed screener/screener rate	26,057	94.31	—		—		—	
Eligible	15,462	59.34	5041	32.60	5307	34.32	5114	33.07
Verified (baseline survey invitation)	5604	36.24	1828	32.62	1902	33.94	1874	33.44
Completed baseline survey^a,b^	4027	71.86	1313	32.60	1400	34.77	1314	32.63

^a^An AAPOR [9] RR6 formula was used for the calculation, with *noncontact* and *other* set to zero.

^b^Includes all cases retained for analysis.

Retention rates for each follow-up survey were calculated for the analytic sample (n=4027; Table 2). Approximately 56.67% (2282/4027) of participants completed all four follow-up surveys, and 19.99% (805/4027) did not complete any of the follow-up surveys. Within each study arm and across arms, retention rates tended to decline as the weeks between baseline and follow-up increased. For example, the retention rate for the 3-week follow-up survey was 74.72% (3009/4027), whereas the retention rate for the 32-week follow-up survey declined to 64.64% (2603/4027). The lowest retention rates in the 3-week and 8-week follow-up surveys were in Arm 1, whereas the lowest retention rates in the 20-week and 32-week follow-up surveys were in Arm 3.

For the 3-week follow-up, Arm 2 had a significantly higher retention rate than Arm 1 (*P*=.01). There were no significant differences between arms at the 8-week and 20-weeks follow-ups. For the 32-week follow-up, Arm 2 had a significantly higher retention rate than Arm 3 (*P*=.03). Arm 1 was significantly more likely than Arm 2 not to complete any follow-up surveys (*P*=.01). Arm 2 was significantly more likely than Arm 3 to complete all follow-up surveys (*P*=.05).

**Table 2:** Retention rates (n=4027).

Follow-up survey	Overall (n=4027)		Arm 1 (n=1313)		Arm 2 (n=1400)		Arm 3 (n=1314)	
	n	%	n	%	n	%	n	%
3-week	3009	74.72	949	72.28	1071	76.50	989	75.27
8-week	2881	71.54	925	70.45	1024	73.14	932	70.93
20-week	2675	66.42	866	65.96	957	68.36	852	64.84
32-week	2603	64.64	846	64.43	933	66.64	824	62.71
Completed no follow-up surveys	805	19.99	292	22.23	256	18.29	257	19.56
Completed all follow-up surveys	2282	56.67	746	56.82	818	58.43	718	54.64

The proportion of participants who completed each survey on the day the invitation was emailed was highest for the baseline survey (56.16%, 3147/5604) and lowest at the 32-week follow-up survey (22.80%, 918/4027), as shown in Table 3. Email and telephone reminders boosted the response rate for all five surveys.

**Table 3:** Percentage of the overall sample completing survey by prompt and data collection point (n=4027).

Percent complete	Baseline	3-week	8-week	20-week	32-week
Day invitation sent	56.2	30.9	31.8	24.1	22.8
Day after invitation sent	5.6	8.9	9.0	8.0	7.9
Single email reminder^a^	1.1–2.1	3.8–7.9	1.4–4.7	2.5–5.6	2.3–7.0
Single phone reminder	0.8	3.0	2.6	3.2	2.9

^a^Four email reminders were sent for each survey.

### Representativeness of the Sample

Multimedia Appendix 1 presents the demographic and smoking history characteristics for the SFTXT analytic sample compared with population estimates from the January, 2011 Current Population Survey (CPS), the most recent survey data available with detailed cigarette smoking information [10]. CPS data are weighted, so counts are not displayed. To create the SFTXT population within CPS, the CPS data were subset down to individuals 18 to 29 years of age who smoked five or more cigarettes per day. The SFTXT sample was predominately female (70.15%, 2825/4027), whereas the CPS population had a more equal distribution of females and males. Compared with the CPS population, the SFTXT sample included more highly educated participants (25.4% vs. 17.3% with a college degree or more), but fewer participants that were employed full-time (39.7% vs. 62.1%). Household income and race/ethnicity characteristics were similar between SFTXT and CPS. In terms of smoking history, the SFTXT sample contained a higher proportion of individuals who tried to quit smoking at least once in the past year (73.8%) compared with the CPS population (60.9%). Average scores on the Heaviness of Smoking Index were higher in the SFTXT sample than in the CPS population, indicating higher nicotine dependence levels among SFTXT participants. Multimedia Appendix 1 also displays demographic and smoking history characteristics within each study arm of the SFTXT sample. These characteristics did not differ significantly by study arm.

## Discussion

### Principal Findings

When designing this study, we reviewed past studies of smoking cessation text messaging interventions to determine what methods had been successfully used in the past. Unfortunately, few studies provided details about study implementation and the effectiveness of strategies that were used to recruit, retain, and manage study participants and reduce fraudulent entry into the study. Our results help to fill these gaps.

Through online advertising, this smoking cessation study resulted in 153,936 visits to the study’s landing page, of whom 27,360 screeners initiated, and 4027 participants were recruited. Antifraud measures prevented, at a minimum, 2448 people from enrolling. Another 3882 respondents were excluded from participating because they did not validate both their email and phone number when sent the verification requests. Of the 4027 participants recruited, 64.64% (2603/4027) were retained across four follow-up waves that extended to 32-weeks post-intervention. Results from our examination of the effectiveness of the enrollment, data collection, and antifraud methods used in this study can be used to inform the methods and procedures developed for future studies. Although the effectiveness of every procedure we used cannot be quantified, we offer the following suggestions based on experience conducting this study.

First, the online advertising and other recruiting efforts were effective in driving more than 150,000 people to the study website, with over half meeting the eligibility criteria. When compared with CPS data, a higher proportion of our sample was female and had higher levels of educational attainment. However, study participants were heavier smokers and had previously tried to quit in the past year, likely because of our eligibility requirement that participants must be at least moderately interested in quitting.

While the total sample was slightly smaller than our target goal of 4248, our retention rates were higher than anticipated, with 56.67% (2282/4027) completing all four follow-up surveys. We had initially planned for approximately 60% attrition over the four waves of follow-up, for a total of 40% completing the final follow-up survey.

The reason for this higher than expected retention rate may have been the incentive of the US $20 Amazon or iTunes gift cards offered for completion of each follow-up survey. Future studies could use smaller incentive amounts to see if they would be equally effective in retaining the sample. We recommend incentivizing only follow-up surveys; offering an incentive for a baseline survey that is recruiting participants through online advertising seemed to invite people to attempt to enter the study more than one time so they could get additional gift cards.

Retention rates differed by arm, with Arm 2 having higher retention rates than Arm 1; this result is not entirely unexpected because Arm 2 received significantly more text messages than Arm 1. However, the amount of communication received does not solely explain differences in retention rates by arm, as Arm 3 received significantly more messages than Arm 2. This finding may indicate a threshold for the amount of messaging that keeps participants engaged in both the intervention and the study. In this case, Arm 1 may have been below that threshold while Arm 3 exceeded that threshold.

Multiple email reminders to encourage participants to complete each survey may have positively affected completion rates for each survey. While we are unable to disentangle the effect of these reminders from the effect of the incentives, our findings suggest that the additional email reminders and the single phone reminder resulted in a small percentage of participants completing a survey, especially for the 32-week follow-up survey, while a single email reminder appears to have prompted 7.0% of the sample to complete that survey. We recommend that researchers who are conducting longitudinal online surveys use multiple email reminders over time to encourage participants to complete each survey.

To receive an invitation to complete each survey, having a correct email address for each participant was necessary. For studies such as this one, we recommend requiring participants to enter their email address two times and programming the data collection instrument to continue only if both email addresses match. However, additional procedures may be needed to ensure that those who are eligible do not have any other barriers to participation. In the overall recruitment process, the largest number of potential participants lost was during phone number and email verification. This number represented a large proportion of the otherwise eligible sample (40.92%, 3882/9486) and, although we sent an email reminding participants to complete the verification process, if their email address was problematic, they would not have received the reminder. Future studies could use both phone and text-based reminders, and should explore other ways to prevent such loss. Without additional research, it is difficult to know all of the possible reasons for so many unverified cases. It is plausible that potential participants did not receive or notice the verification email and/or text, or felt it was too burdensome to go through that process. Others may simply have forgotten. Regardless of the reasons for their dropout, these individuals were known to be eligible and had provided contact information. Additional research could help to determine whether email or phone information is inaccurate, or if reminders to verify (or instructions emphasizing the simplicity of the verification procedures) can help to reduce the number of participants who do not complete the process. In addition, researchers should be mindful of such instances during the recruitment phase, because this type of sample loss incurs unnecessary costs to the recruitment effort.

Previous Internet studies of smoking cessation have found some respondents to be fraudulent [2]. The antifraud procedures we established allowed us to identify more than 5000 cases that would have been included in the dataset otherwise. Thus, we were able to screen out participants who may have continued to provide bad or duplicate data, and enhance the overall quality of the dataset. The results of this study add further evidence to growing literature that suggests antifraud measures can help deter some respondents who otherwise might have provided less complete or invalid data [8].

### Limitations

Compared with CPS data, our sample was more likely to be female, younger, more educated, heavier smokers, and more likely to have tried quitting in the last year than the general population of smokers 18 to 29 years of age. CPS data did not ask how motivated smokers were to quit, leaving us unable to determine if our sample was more motivated than the general population of smokers 18 to 29 years of age; being moderately interested in quitting was a study requirement. Consequently, the results cannot be generalized beyond this specific sample. Our findings are limited, because we did not design our study to determine the effectiveness of individual study procedures (eg, email reminders, phone reminder, incentives) in retaining participants. Future studies could be designed to conduct experiments to compare these methods.

### Comparison with Prior Work

Our results detail specific methods used to conduct an online longitudinal study of a text-based smoking cessation intervention. Other studies have reported on outcomes, but to date, no other studies provide detailed information about the effectiveness of the operational methods used to conduct the studies.

### Conclusions

The methods described here helped the project team to recruit an overall sample of more than 4000 smokers for the study. Antifraud measures helped to catch participants who tried to collect multiple incentives or were unable to verify their phone numbers or email addresses. This step may have resulted in loss of potentially eligible sample members, but may have contributed to higher retention rates than we originally projected. This tradeoff is a lesson for future studies. Additional research should explore more efficient ways to conduct such verification without risking the loss of so many potential respondents.

## Appendix

Multimedia Appendix 1 Demographics of SFTXT analytic sample compared with Census estimates, overall and by study arm (n=4027).

## Abbreviations

AAPOR: American Association for Public Opinion Research
CPS: Current Population Survey
IP: Internet Protocol
NCI: National Cancer Institute
SFTXT: SmokefreeText
SMS: short message service
