# Supplementary material for: The SmokefreeTXT (SFTXT) Study: Web and Mobile Data Collection to Evaluate Smoking Cessation for Young Adults
Source: JMIR Res Protoc. 2016 Jun 27;5(2):e134. doi: 10.2196/resprot.5653 (PMC4940604; doi:10.2196/resprot.5653)
Supplement: Multimedia Appendix 1 [file resprot_v5i2e134_app1.pdf]

Multimedia Appendix 1. Demographics of SFTXT analytic sample compared with Census estimates, overall and by study arm (n=4027).

Characteristic	Census estimates for the QuitTXT eligible population[a]	Overall for the analytic sample	Analytic sample – Arm 1	Analytic sample – Arm 2	Analytic sample – Arm 3
**Sex, % (n)**					
Male	56.1	29.8 (1200)	29.6 (389)	29.4 (412)	30.4 (399)
Female	43.9	70.2 (2825)	70.4 (923)	70.6 (988)	69.6 (914)
**Age (years), % (n)**					
18–21	11.0	20.8 (839)	19.6 (257)	21.0 (294)	21.9 (288)
22–25	39.2	34.9 (1405)	35.2 (462)	35.3 (494)	34.2 (449)
26–29	49.8	44.3 (1783)	45.2 (594)	43.7 (612)	43.9 (577)
**Race/Ethnicity, % (n)**					
Non-Hispanic white	76.7	73.7 (2967)	72.9 (957)	73.5 (1029)	74.7 (981)
Non-Hispanic black	7.5	9.0 (362)	8.5 (112)	9.2 (129)	9.2 (121)
Non-Hispanic other	5.7	7.7 (312)	8.4 (110)	7.4 (104)	7.5 (98)
Hispanic	10.1	9.6 (386)	10.2 (134)	9.9 (138)	8.7 (114)
**Education, % (n)**					
Less than high school	12.2	5.6 (225)	5.7 (74)	5.3 (74)	5.9 (77)
High school/GED	37.0	23.6 (946)	22.7 (296)	24.2 (337)	23.9 (313)
Some college	32.6	45.4 (1819)	46.3 (603)	45.3 (632)	44.6 (584)
College degree or more	18.1	25.4 (1018)	25.3 (330)	25.2 (352)	25.6 (336)
**Income, % (n)**					
Less than $35,000	63.4	57.3 (2306)	57.0 (748)	57.0 (798)	57.9 (760)
$35,000 and $70,000	25.0	23.7 (954)	25.2 (331)	23.3 (326)	22.6 (297)
Over $70,000	11.6[b]	7.6 (306)	7.5 (98)	7.9 (111)	7.4 (97)
**Employment status, % (n)**					
Employed part time	16.3	22.3 (899)	22.6 (297)	21.9 (306)	22.5 (296)

Characteristic	Census estimates for the QuitTXT eligible population[a]	Overall for the analytic sample	Analytic sample – Arm 1	Analytic sample – Arm 2	Analytic sample – Arm 3
Employed full time	63.0	39.7 (1597)	38.9 (511)	40.5 (567)	39.5 (519)
Not currently employed	20.8	38.0 (1530)	38.5 (505)	37.6 (527)	37.9 (498)
**Number of quit attempts in last 12 months**					
Has not tried to quit	38.9	26.2 (1054)	27.7 (363)	26.3 (367)	24.7 (324)
One	14.0	19.1 (766)	17.2 (226)	20.0 (279)	19.9 (261)
Two or more	47.1	54.7 (2200)	55.1 (723)	53.8 (751)	55.4 (726)
Got help from family or friends to stop smoking, n (%)	20.1	11.9 (476)	12.3 (160)	13.2 (183)	10.2 (133)
Number of cigarettes per day in past 30 days, mean (SD)	9.7 (6.9)	12.3 (8.3)	12.4 (8.1)	12.3 (8.6)	12.3 (8.3)
Heaviness of Smoking Index (0-6 scale), mean (SD)[c]	1.6 (1.4)	3.7 (1.4)	3.6 (1.4)	3.7 (1.4)	3.7 (1.4)

[a]Census Current Population Survey: January 2011. Standard errors for estimate are not shown as the information required to develop standard errors is restricted.
[b]Census category stops at $75,000.
[c]Scores range from 0 to 6, with 1–2 = very low dependence; 3 = low to moderate dependence; 4 = moderate dependence; and 5+ = high dependence (Heatherton et al [11]).
